# Core fucosylation regulates alveolar epithelial cells senescence through activating of transforming growth factor-β pathway in pulmonary fibrosis

**DOI:** 10.18632/aging.205036

**Published:** 2023-09-18

**Authors:** Yu Jiang, Zhongzhen Wang, Jinying Hu, Wei Wang, Na Zhang, Lili Gao

**Affiliations:** 1 Department of Respiratory Medicine, The First Affiliated Hospital of Dalian Medical University, Dalian, China; 2 Department of Cardiology, Institute of Cardiovascular Diseases, The First Affiliated Hospital of Dalian Medical University, Dalian, China; 3 Department of Nephrology, Affiliated Xinhua Hospital of Dalian University, Dalian, China

**Keywords:** cell senescence, idiopathic pulmonary fibrosis, core fucosylation, alveolar epithelial cell

## Abstract

Idiopathic pulmonary fibrosis (IPF), a fatal disorder associated with aging, has a terrible prognosis. However, the potential causes of IPF remain a riddle. In this study, we designed to explore whether the modification of the core fucosylation (CF) can ameliorate pulmonary fibrosis by targeting alveolar epithelial cells (AECs) senescence. First, we verified that cellular senescence occurs in the bleomycin-induced lung fibrosis mice models and CF modifications accompanying senescent AECs in pulmonary fibrosis. Next, both gain- and loss- of function research on CF were performed to elucidate its role in promoting AECs senescence and triggering pulmonary fibrosis *in vitro*. Notably, using alveolar epithelial cell-specific FUT8 conditional knockout mouse models, however, inhibition of cellular senescence by deleting the FUT8 gene could attenuate pulmonary fibrosis *in vivo*. Finally, blocking the CF modification of transforming growth factor -β type I receptor (TGF-βR I) could reduce the activation of downstream transforming growth factor -β (TGF-β) pathways in AECs senescence both *in vivo* and *in vitro*. This study reveals that CF is a crucial interventional target for the treatment of pulmonary fibrosis. Blocking CF modification contributes importantly to inhibiting AECs senescence resulting in pulmonary fibrosis lessen.

## INTRODUCTION

IPF is a chronic, progressive, and fatal idiopathic fibrotic disorder of unknown etiology with a poor prognosis that only has a 2 to 3 years median survival following diagnosis [1, 2]. Pathological features of IPF are characterized by a destroyed alveolar architecture, excessive extracellular matrix production, and are associated with a histopathological and radiological pattern of usual interstitial pneumonia [3, 4]. IPF occurs primarily in old people, while the incidence and prevalence increase strongly with age [3, 5]. Various factors have been associated with the etiology of IPF, such as cigarette smoking, ecological exposures, gastroesophageal reflux, and genetic factors; of these, age is the most compelling [2, 6]. To date, although nintedanib and pirfenidone treatment is recommended as conditional recommendations for patients with IPF, no novel pharmacological therapies have received strong recommendations [7]. Therefore, elucidating the potential mechanisms of IPF and then generating innovative interventions are urgent clinical needs.

Cell senescence can be established as a cell state triggered by various physiological processes, characterized by an irreversible cell-cycle arrest in response to numerous stressors [8]. As one of nine candidate hallmarks of aging, cell senescence is associated with aging-related diseases [9, 10]. To date, the negative impacts of cell senescence have been linked to the causative potency of disorders affecting various organ systems, such as pulmonary disease [11]. Furthermore, cellular senescence regulates the balance between fibrosis and healing in regenerative processes by promoting the production of factors for tissue remodeling [12–14]. Additionally, evidence research suggested that AECs senescence may be a characteristic of lung fibrosis through PTEN/NF-κB pathway [15]. Suppression of alveolar type II cells senescence is connected to a reduction in lung fibrosis and TGF-β I in part induced alveolar type II cells senescence [16, 17]. As a result, elucidation of the mechanisms underlying AECs senescence and activated molecular biological effects is crucial for creating future IPF therapies.

Glycosylation, as one of the most common and complex post-translational modifications, has a profound impact on proteomics [18]. Based on the fundamental influence of protein glycosylation on numerous biological processes such as differentiation, cell maturation, and so on, CF is catalyzed by α1,6-fucosyltransferase which is the only enzyme in mammals that also plays a prominent role [19, 20]. FUT8 is responsible for adding fucosyl moiety to glycoproteins by α1,6 linkage to produce the modification of CF [21]. Previous studies have demonstrated a strong association between FUT8 and fibrotic disorders [22–24]. Mesenchymal Stem Cell-derived extracellular vesicles act on CF of several proteins via inhibiting the profibrotic pathway in renal interstitial fibrosis [22]. Blocking CF attenuated peritoneal fibrosis is performed through the deactivation of the PDGF and TGF-β signaling pathways [23]. Meanwhile, CF regulates pericyte transition and may suppress lung fibrosis [24]. These results indicated that CF may be a novel therapeutic target of IPF. However, the function of CF and its potential processes remain to be revealed. In this study, using cultured MLE_12_ cells and alveolar epithelial cell-specific FUT8 conditional knockout (CKO) mouse models, we show that CF mediates the modification of TGF-βR I which is a critical module in the TGF-β pathway and regulates cell senescence induced by bleomycin (BLM) *in vivo* and *in vitro*. Inhibition of cellular senescence by eliminating the FUT8 gene could attenuate pulmonary fibrosis. These results reveal that activated CF modification leads to cellular senescence in lung disorders with fibrosis.

## RESULTS

### Cellular senescence occurs in the BLM-induced pulmonary fibrosis mice model

**Figure 1:**
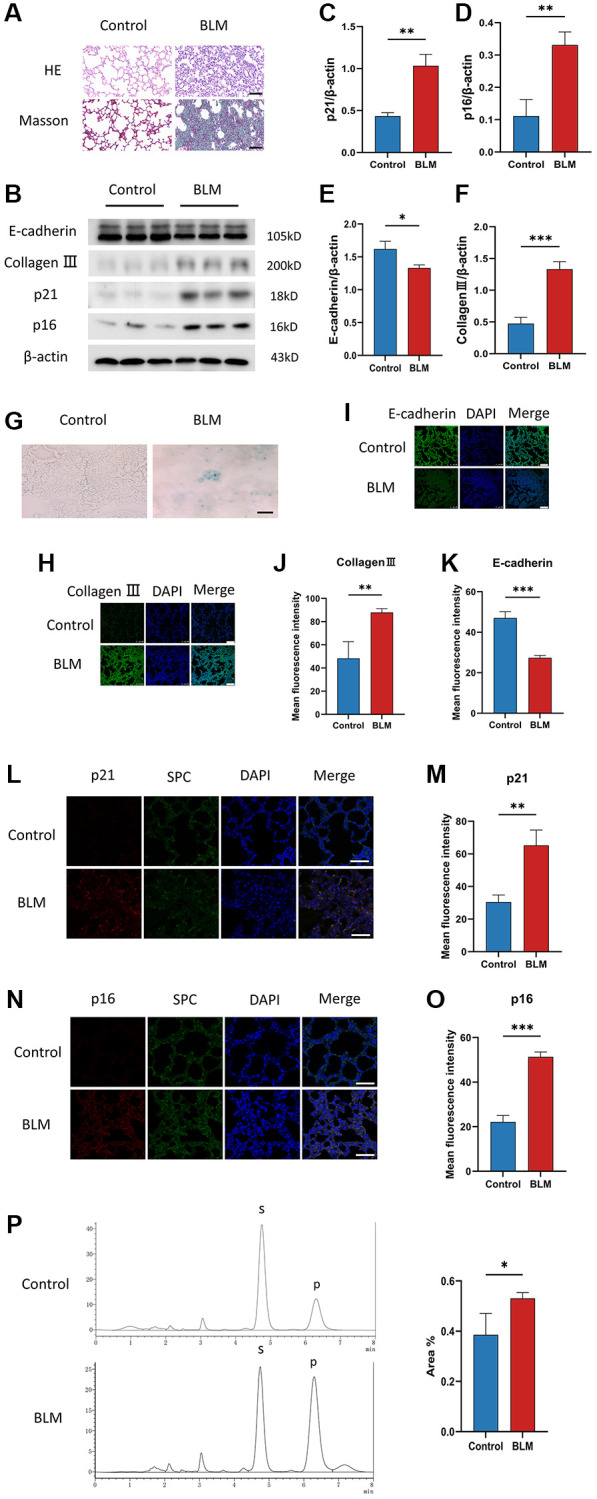
**Cellular senescence occurs in BLM-induced lung fibrosis mice model.** (**A**) HE and Masson staining reveal representative pictures of different lung tissue sections after 21 days of BLM treatment. Scale bar, 100 μm. (**B**–**F**) Western blotting using anti-E-cadherin, anti-collagen III, anti-p21, anti-p16, and anti-β-actin antibodies in mice treated with BLM or saline. The grayscale evaluations of the bands were adjusted to be equal to β-actin. Data were presented as the mean ± SD. ^*^P < 0.05 compared to control (Unpaired *t*-test). ^**^P < 0.01 compared to control (Unpaired *t*-test). ^***^P < 0.001 compared to the control (Unpaired *t*-test). (**G**) SA-β-gal staining reveals representative images of different lung tissue sections. Scale bar, 100 μm. (**H**–**K**) The protein levels of collagen III and E-cadherin in different lung tissues were measured via immunofluorescence technique. DAPI was used to counterstain the nuclei. Scale bar 100 μm. Data were presented as the mean ± SD. ^**^P < 0.01 compared to control (Unpaired *t*-test). ^***^P < 0.001 compared to the control (Unpaired *t*-test). (**L**–**O**) Representative immunofluorescence shows colocalization of p21^WAF1^ or p16^ink4a^ (Red) and SPC (Green) in lung tissue. DAPI was used to counterstain the nuclei. Scale bar, 50 μm. Data were presented as the mean ± SD. ^**^P < 0.01 compared to control (Unpaired *t*-test). ^***^P < 0.001 compared to the control (Unpaired *t*-test). (**P**) High performance liquid chromatography shows FUT8 activity in mice. Data are the percentage of the area of P divided by the area of S+P and presented as the mean ± SD. P is the fucosylation product, and S is the peptide substrate. ^*^P < 0.05 compared to the control (Unpaired *t*-test).

To investigate the appearance of cellular senescence in lung fibrosis, we used C57/BL6 mice to construct a BLM-induced pulmonary fibrosis mice model. In the lung tissue of BLM-induced mice, Masson and HE staining exhibited that accumulation of collagen fibers and destruction of the alveolar structure (Figure 1A). The levels of p16^ink4a^ and p21^WAF1^ are two senescent mediators detected by western blotting, were increased in lung tissue of BLM-induced mice compared to saline-injected mice (Figure 1B–1D), just as the level of the SA-β-gal staining, which is a sign of cellular senescence (Figure 1G). Meanwhile, BLM treatment of mice significantly decreased the E-cadherin protein levels and increased collagen III protein levels (Figure 1B, 1E, 1F). Surfactant protein C (SPC) is an alveolar epithelial cell specific marker [25]. We detected p16^ink4a^ or p21^WAF1^ protein expression in lung tissue using immunofluorescence co-localization of p16^ink4a^, SPC, or p21^WAF1^, SPC. Similarly, immunofluorescence staining further showed that the fibrotic markers collagen III, and the senescent markers p16^ink4a^, p21^WAF1^ were overexpression in lung tissue of BLM-induced mice, along with decreased E-cadherin expression (Figure 1H–1O). In addition, we found that the FUT8 enzyme activity in the lung tissue of BLM-induced mice was increased compared to the control group (Figure 1P). Together, these results confirmed that cellular senescence existed and FUT8 enzyme activity was overexpression in BLM-induced lung fibrosis.

### CF modifications accompanying AECs senescence in pulmonary fibrosis

**Figure 2:**
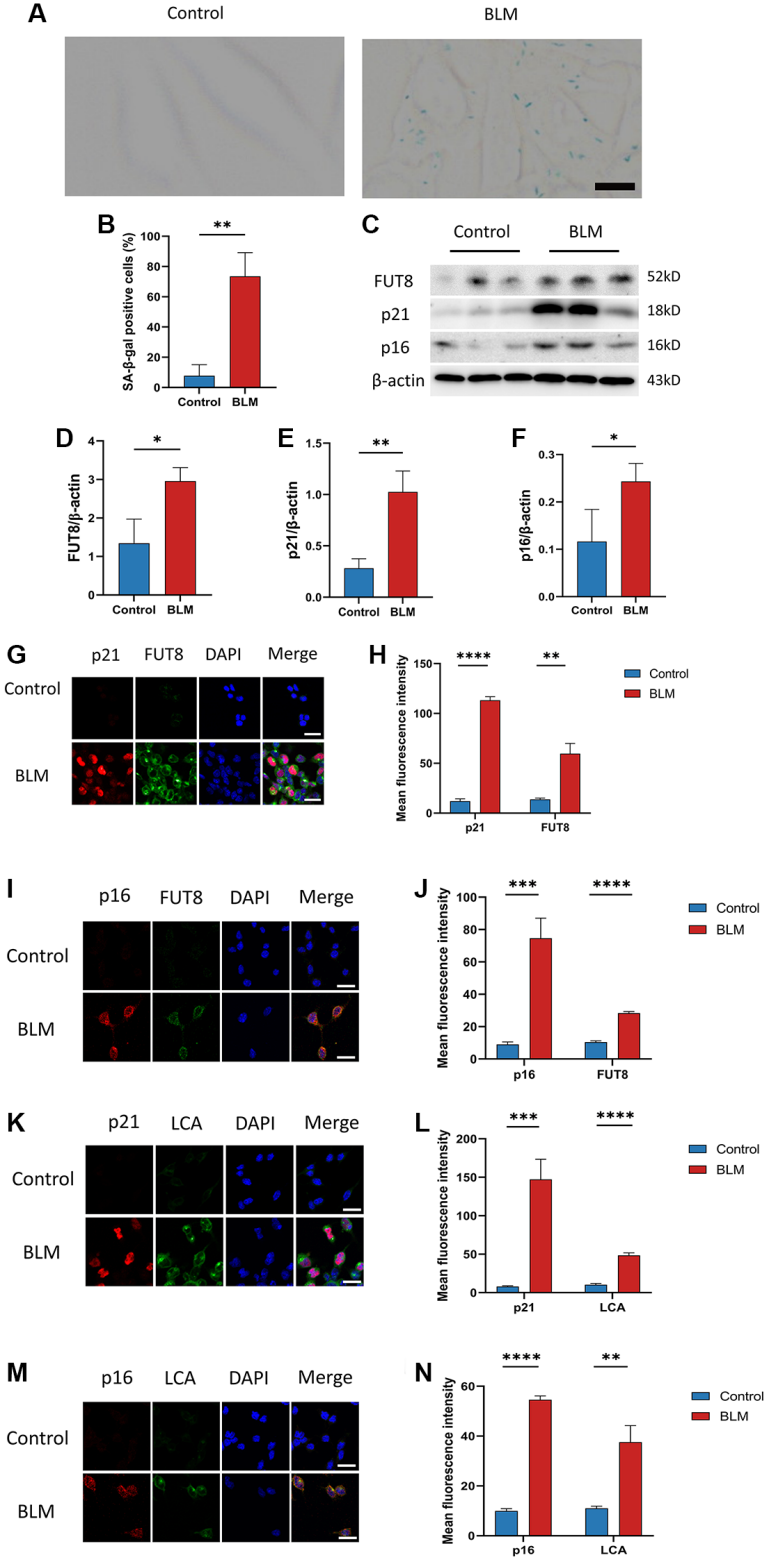
**CF modifications accompanying AECs senescence in pulmonary fibrosis.** (**A**, **B**) SA-β-gal staining shows representative images of MLE_12_ cells after 72 hours of BLM treatment. Scale bar, 10μm. Data were presented as the mean ± SD. ^**^P < 0.01 compared to control (Unpaired *t*-test). (**C**–**F**) Western blotting using anti-FUT8, anti-p21, anti-p16, and anti-β-actin antibodies in MLE_12_ cells treated with BLM or saline. The grayscale evaluations of the bands were adjusted to be equal to β-actin. Data were presented as the mean ± SD. ^*^P < 0.05 compared to control (Unpaired *t*-test). ^**^P < 0.01 compared to control (Unpaired *t*-test). (**G**–**J**) Representative immunofluorescence shows colocalization of p21^WAF1^ or p16^ink4a^ (Red) and FUT8 (Green) in MLE_12_ cells. DAPI was used to counterstain the nuclei. Scale bar, 25 μm. Data were presented as the mean ± SD. ^**^P < 0.01 compared to control (Unpaired *t*-test). ^***^P < 0.001 compared to control (Unpaired *t*-test). ^****^P < 0.0001 compared to control (Unpaired *t*-test). (**K**–**N**) Immunofluorescence shows colocalization of p21^WAF1^ or p16^ink4a^ (Red) and LCA (Green) in MLE_12_ cells treated with BLM. DAPI was used to counterstain the nuclei. Scale bar, 25 μm. Data were presented as the mean ± SD. ^**^P < 0.01 compared to control (Unpaired *t*-test). ^***^P < 0.001 compared to control (Unpaired *t*-test). ^****^P < 0.0001 compared to control (Unpaired *t*-test).

Although FUT8 expression was increased in BLM-induced mice lungs, the relationship between CF modifications and AECs senescence was still unknown. We detected changes in CF modifications in BLM-induced MLE_12_ cells. On the one hand, as expected, SA-β-gal staining was found mainly in BLM-induced MLE_12_ cells, while it was hardly seen in control MLE_12_ cells (Figure 2A, 2B). Subsequently, western blotting suggested that p16^ink4a^ and p21^WAF1^ proteins expressions were higher in BLM-induced MLE_12_ cells than in the control group, indicating that senescence occurred in MLE_12_ cells (Figure 2C, 2E, 2F). On the other hand, a significantly increased FUT8 expression was observed in BLM-induced MLE_12_ cells compared with the control group (Figure 2C, 2D). Moreover, the spatial distribution of FUT8 and senescent indicators p16^ink4a^ or p21^WAF1^ in MLE_12_ cells was examined using immunofluorescence. The expressions of p16^ink4a^ or p21^WAF1^ have the parallel trend with FUT8 expression in BLM- induced or control MLE_12_ cells (Figure 2G–2J). In addition, lens culinaris agglutinin (LCA) can recognize α1-6 fucosylation on N-gly-cans [26], so we also examined whether CF occurs during cellular senescence by using fluorescein-labeled LCA. The effects of BLM-induced p16^ink4a^ or p21^WAF1^ with LCA expressions in AECs senescence are subsequently validated by double immunostaining of MLE_12_ cells (Figure 2K–2N). In conclusion, these results revealed that AECs senescence was associated with CF modifications in pulmonary fibrosis, which implied that CF could be a critical interventional target to modulate cellular senescence consulting in pulmonary fibrosis.

### Silencing CF modifications with FUT8 siRNA rescues AECs from senescence and attenuates BLM-induced lung fibrosis

**Figure 3:**
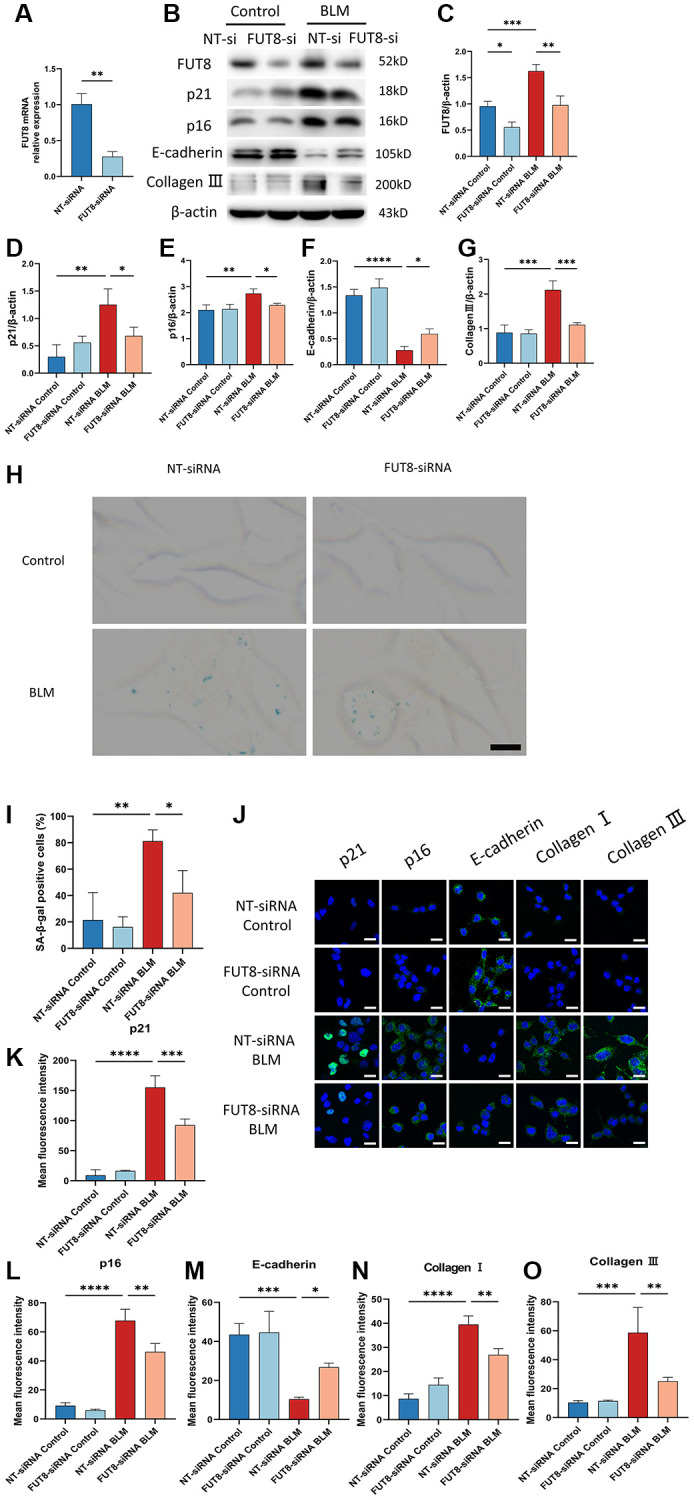
**Silencing CF modifications with FUT8 siRNA rescues AECs from senescence and attenuates BLM-induced lung fibrosis.** NT siRNA or FUT8 siRNA was used to transfect MLE_12_ cells, which were subsequently treated or untreated with BLM for 48 hours. (**A**) To test the gene silencing effect, we use real-time polymerase chain reaction (RT-PCR) to show FUT8 mRNA expression in MLE_12_ cells transfected with NT siRNA or FUT8 siRNA. ^**^P < 0.01 compared to NT siRNA (Unpaired *t*-test). (**B**–**G**) Western blotting using anti-FUT8, anti-p21, anti-p16, anti-E-cadherin, anti-collagen III, and anti-β-actin antibodies in MLE_12_ cells treated in different ways. Densitometric analyses of the bands were adjusted to be equal to β-actin. Data were presented as the mean ± SD. ^*^P < 0.05. ^**^P < 0.01. ^***^P < 0.001. ^****^P < 0.0001. (**H**, **I**) SA-β-gal staining reveals indicative pictures of different treated MLE_12_ cells. Scale bar, 10 μm. Data were presented as the mean ± SD. ^*^P < 0.05. ^**^P < 0.01. (**J**–**O**) E-cadherin, p21^WAF1^, p16^ink4a^, collagen I, and collagen III expressions were measured via the immunofluorescence technique. Scale bar 25 μm. Data were presented as the mean ± SD. ^*^P < 0.05. ^**^P < 0.01. ^***^P < 0.001. ^****^P < 0.0001.

Next, to determine the role of CF modifications in AECs senescence, nontarget siRNA (NT siRNA) or FUT8 siRNA (FUT8 siRNA) were used to transfect MLE_12_ cells, which were subsequently treated or untreated with BLM. RT-PCR analysis confirmed that MLE_12_ cells treated with FUT8 siRNA significantly decreased FUT8 levels. (Figure 3A). Western blotting showed that the levels of FUT8, p16^ink4a^, and p21^WAF1^ were significantly increased in transfected with nontarget siRNA of BLM-induced MLE_12_ cells while silencing FUT8 ameliorated BLM-stimulated increased in FUT8, p16^ink4a^ and p21^WAF1^ protein expression (Figure 3B–3E), as well as in SA-β-gal activity (Figure 3H, 3I). Consistent with this, immunofluorescence staining similarly showed that knockdown FUT8 with FUT8 siRNA decreased the expression of p16^ink4a^ and p21^WAF1^ in BLM-induced MLE_12_ cells compared with transfected nontarget siRNA in BLM-induced MLE_12_ cells (Figure 3J–3L). In particular, western blotting (Figure 3B, 3F, 3G) and immunofluorescence staining (Figure 3J, 3M–3O) suggested that FUT8 siRNA attenuated the increased collagen III and collagen I proteins expressions mediated by BLM, and restores BLM-mediated suppression of E-cadherin. Together, these findings indicated that CF plays a critical role in BLM-induced AECs senescence. Blocking CF modifications may obstruct the process of AECs senescence and partially ameliorate lung fibrosis.

### Blocking CF modification of TGF-βR I alleviates activation of TGF-β signaling pathway in BLM-induced AECs senescence

**Figure 4:**
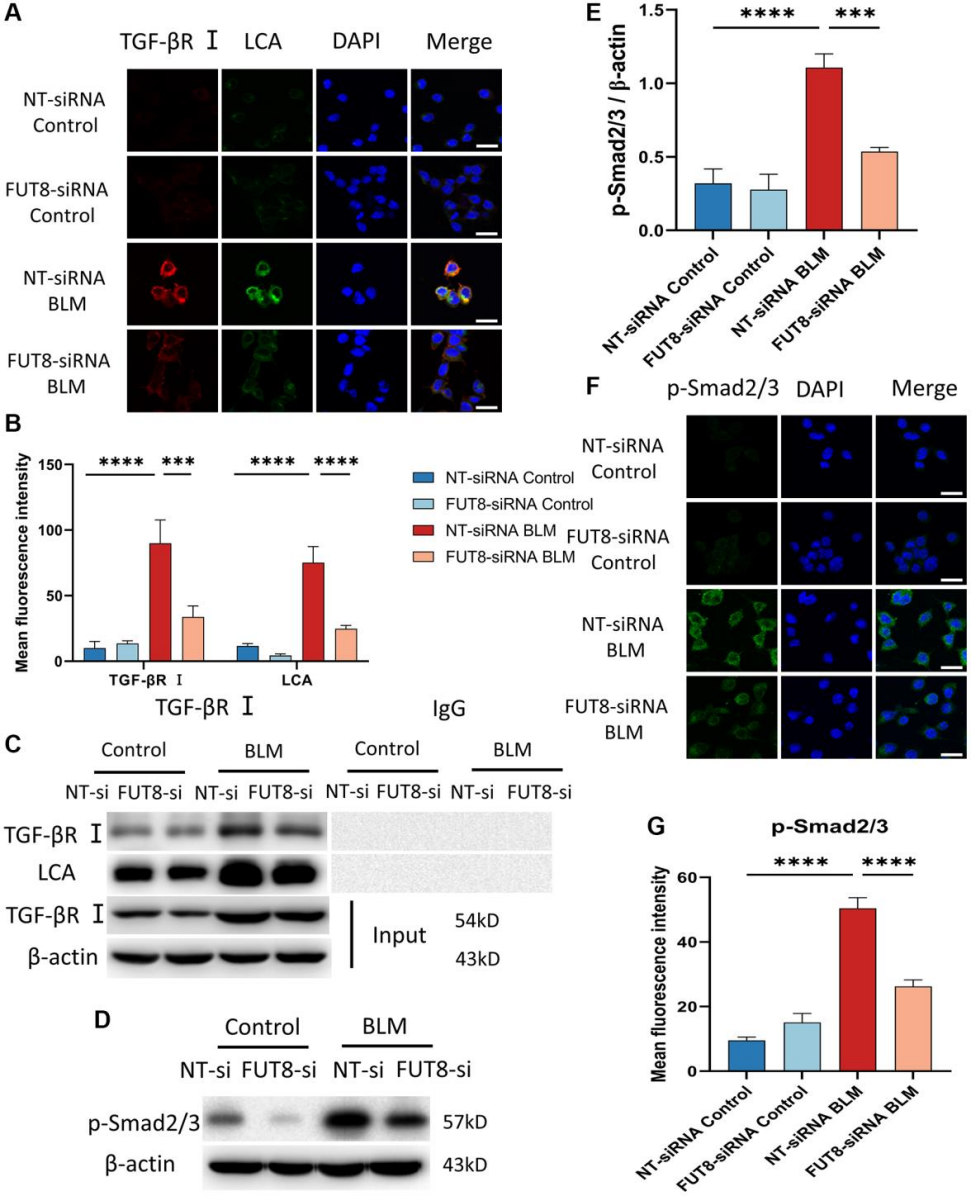
**Blocking CF modification of TGF-βR I alleviates activation of TGF-β signaling pathway in BLM-induced AECs senescence**. (**A**, **B**) Representative immunofluorescence shows the colocalization of TGF-βR I (Red) and LCA (Green) in different treatments with MLE_12_ cells. DAPI was used to counterstain the nuclei. Scale bar, 25 μm. Data were presented as the mean ± SD. ^***^P < 0.001. ^****^P < 0.0001. (**C**) Immunoprecipitation of TGF-βR I with LCA was tested by immunoblotting analysis. (**D**, **E**) Western blot using anti-p-Smad2/3 and anti-β-actin antibodies. Densitometric analyses of the bands were adjusted to be equal to β-actin. Data were presented as the mean ± SD. ^***^P < 0.001. ^****^P < 0.0001. (**F**, **G**) The p-Smad2/3 expression in different groups was measured via the immunofluorescence technique. DAPI was used to counterstain the nuclei. Scale bar 25 μm. Data were presented as the mean ± SD. ^****^P < 0.0001.

Relative studies showed that cellular senescence involves multiple signaling pathways that contribute to the development of diseases. Bronchial epithelial cell-derived extracellular vesicle inhibits TGF-β regulated induction of lung epithelial cell senescence in IPF [27]. Cellular senescence is regulated by the PI3K/FOXO and TGF-β/Smad signaling pathways during the endolymphatic sac of the mesonephros and the inner ear [28]. Based on these fundamental data, we elucidate whether the mechanisms underlying AECs senescence could be modulated by CF. TGF-βR I was recognized with red fluorescent, while LCA was specifically recognized with green fluorescent in immunofluorescence staining, suggesting that the fluorescence intensity of TGF-βR I and LCA both increased in BLM-induced MLE_12_ cells transfected with nontarget siRNA, however, silencing FUT8 reduced BLM-mediated increased in TGF-βR I and LCA expression (Figure 4A, 4B). To further investigate that TGF-βR I, a key protein in the TGF-β pathway, can be modified by CF, we used immunoprecipitation to detect the expression of LCA. The results revealed that the level of LCA was higher in BLM-induced MLE_12_ cells than in the control group transfected with non-target siRNA, but LCA expression was decreased in BLM-induced MLE_12_ cells transfected with FUT8 siRNA compared to non-target siRNA (Figure 4C). Moreover, significant differences in the phosphorylation level of Smad2/3 were observed between BLM-induced MLE_12_ cells transfected with FUT8 siRNA and nontarget siRNA in western blotting and immunofluorescence staining (Figure 4D–4G). Knockdown FUT8 decreased the phosphorylation of Smad2/3 (p-Smad2/3) protein expression. According to the findings, blocking CF modification of TGF-βR I could reduce the activation of the downstream pathways of TGF-β in AECs senescence.

### Inhibition of TGF-βR I activity with EW-7197 abrogates overexpression of CF modification induced AECs senescence

**Figure 5:**
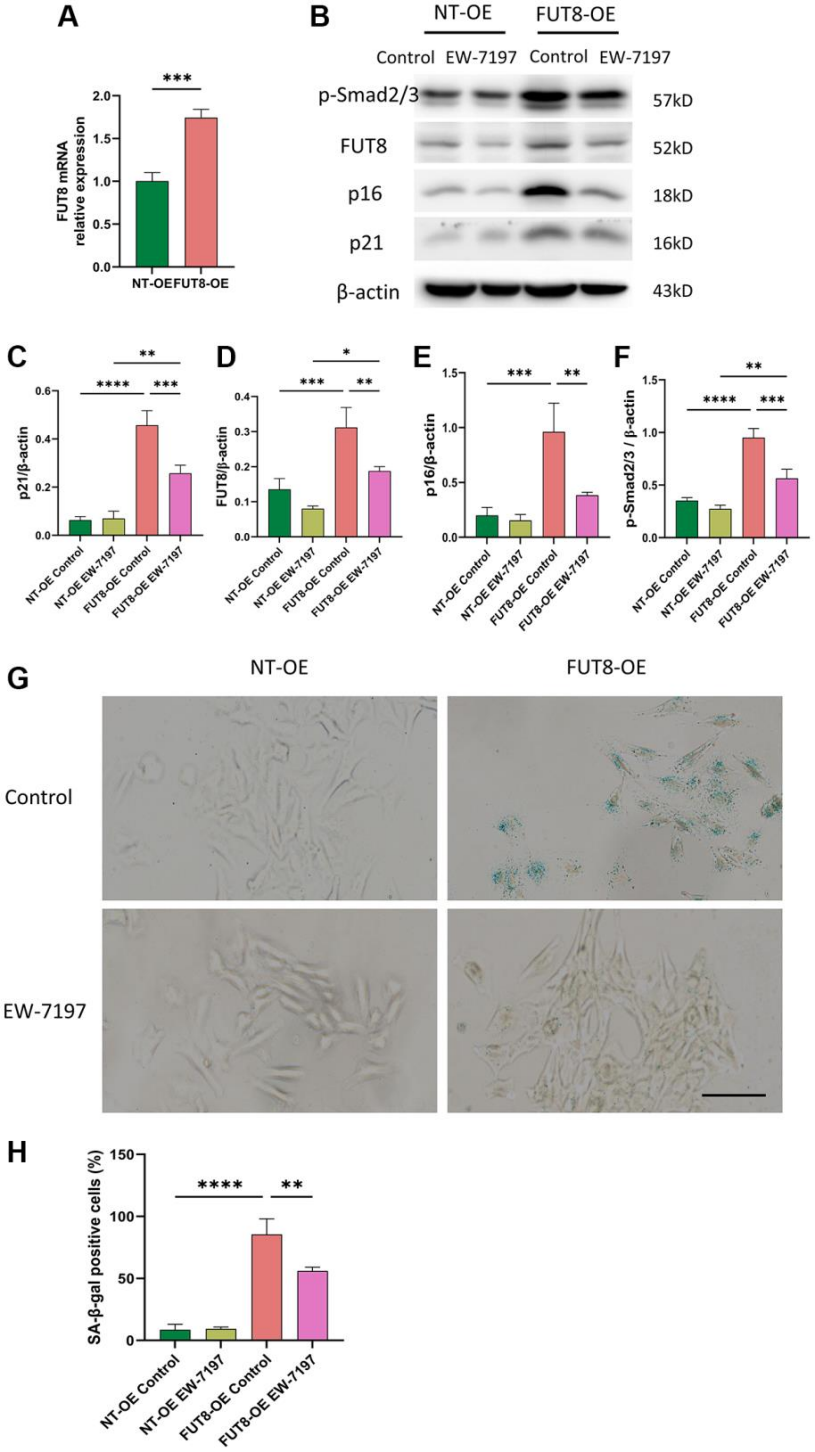
**Inhibition of TGF-βR I activity with EW-7197 abrogates overexpression of CF modification induced AECs senescence**. MLE_12_ cells were infected with an adenovirus expression vector overexpressing FUT8 and then stimulated with EW-7197 for 6 hours. (**A**) To test the effect of gene overexpression, we used RT-PCR to show FUT8 mRNA expression in MLE_12_ cells infected with FUT8-OE or NT-OE. ^***^P < 0.001 compared with NT OE group (Unpaired *t*-test). (**B**–**F**) Western blotting using anti-p-Smad2/3, anti- FUT8, anti-p16, anti-p21 and anti-β-actin antibodies. Densitometric analyses of the bands were adjusted to be equal to β-actin. Data were presented as the mean ± SD. ^*^P < 0.05. ^**^P < 0.01. ^***^P < 0.001. ^****^P < 0.0001. (**G**, **H**) SA-β-gal staining shows indicative pictures. Scale bar, 100 μm. Data were presented as the mean ± SD. ^**^P < 0.01. ^****^P < 0.0001.

EW-7197, a small molecule ALK5 inhibitor, represents a potent TGF-βR inhibitor [29]. To further determine whether CF modifications induce AECs senescence by activating the TGF-β signaling pathway, MLE_12_ cells were infected with adenovirus expression vector overexpressing FUT8 (FUT8 OE) or nontarget (NT OE) in the presence or absence of EW-7197. RT-PCR analysis confirmed that MLE_12_ cells infected with the adenovirus vector successfully induced the overexpression of FUT8 mRNA (Figure 5A). As shown in western blotting, FUT8 OE enhanced Smad2/3 phosphorylation, along with the expression of FUT8, p16^ink4a^ and p21^WAF1^ proteins (Figure 5B–5F), which is linked to an increase in SA-β-gal activity (Figure 5G, 5H). Furthermore, treatment of MLE_12_ cells infected with FUT8 OE and then treated with EW-7197 significantly decreased phosphorylation of Smad2/3, FUT8, p16^ink4a^, and p21^WAF1^ expression (Figure 5B–5F), along with the positive cells in SA-β-gal staining (Figure 5G, 5H). Collectively, these results elucidated that CF modification may contribute to AECs senescence via at least activating the TGF-β signaling pathway.

### CKO mice inhibit BLM-induced cellular senescence and ameliorate lung fibrosis *in vivo*

**Figure 6:**
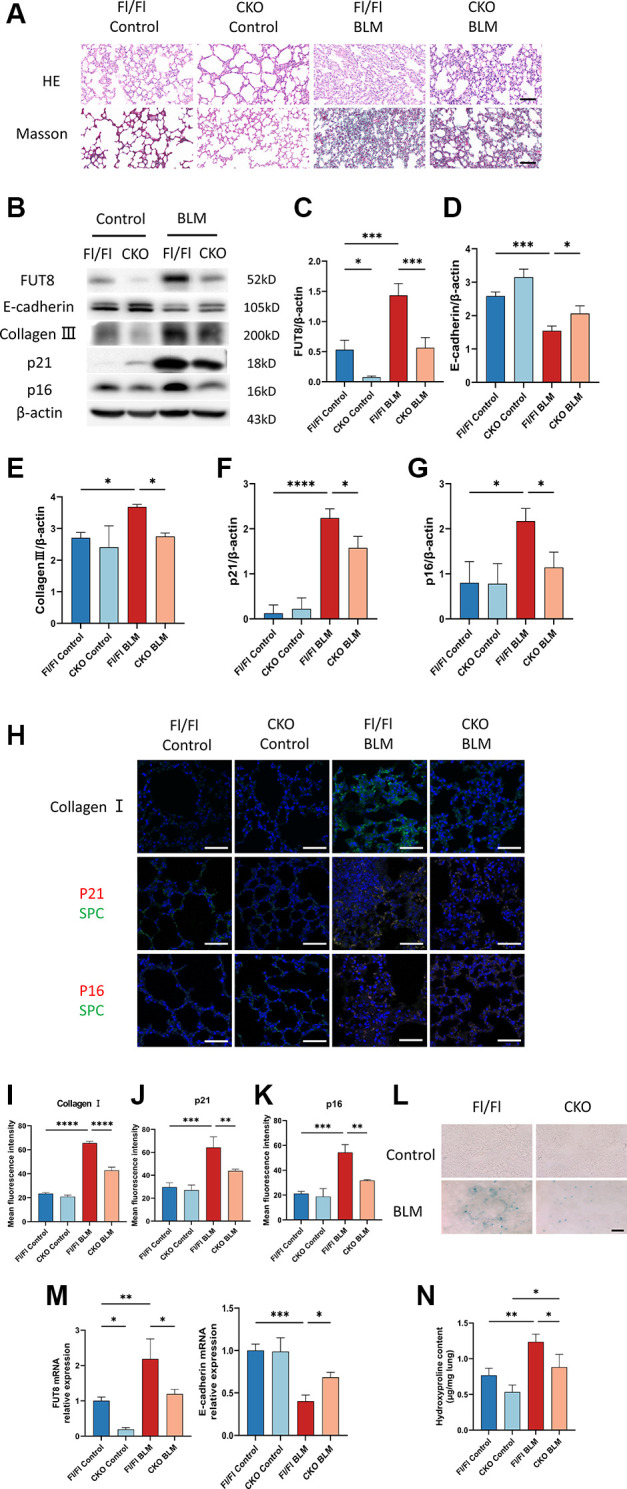
**CKO mice inhibit BLM-induced cellular senescence and ameliorate lung fibrosis *in vivo*.** (**A**) CKO mice and Fl/Fl mice were treated with BLM for 21 days. HE and Masson staining reveal representative images of different lung tissue sections. Scale bar, 100 μm. (**B**–**G**) Western blotting using anti-FUT8, anti-E-cadherin, anti-collagen III, anti-p21, anti-p16, and anti-β-actin antibodies. Densitometric analyses of the bands were adjusted to be equal to β-actin. Data were presented as the mean ± SD. ^*^P < 0.05. ^***^P < 0.001. ^****^P < 0.0001. (**H**–**K**) The protein levels of collagen I in different mice were measured via immunofluorescence technique. Representative immunofluorescence shows colocalization of p21^WAF1^ or p16^ink4a^ (Red) and SPC (Green) in the lung tissue of different mice. Scale bar 50 μm. Data were presented as the mean ± SD. ^**^P < 0.01. ^***^P < 0.001. ^****^P < 0.0001. (**L**) SA-β-gal staining reveals indicative pictures of different lung tissue sections. Scale bar, 100 μm. (**M**) RT-PCR revealed FUT8 and E-cadherin mRNAs expression in different groups. Data were presented as the mean ± SD. ^*^P < 0.05. ^**^P < 0.01. ^***^P < 0.001. (**N**) Hydroxyproline content of lung tissues. Data were presented as the mean ± SD. ^*^P < 0.05. ^**^P < 0.01.

We verified that the BLM-induced mouse model increased collagen and decreased E-cadherin expression leading to fibrosis. To explore whether knockout of the FUT8 gene in AECs will rescue AECs from senescence and ameliorate pulmonary fibrosis *in vivo*, we devised an *in vivo* assay with alveolar epithelial cell-specific FUT8 conditional knockout (FUT8^flox/flox^; Sftpc^CRE^, CKO) mice in our previous study and chosen FUT8^flox/flox^ (Fl/Fl) mice as the control. HE and Masson staining showed that compared with BLM-induced Fl/Fl mice, the damage of alveolar structure reduced and collagen deposition decreased in BLM-induced CKO mice (Figure 6A). Western blotting suggested that BLM-induced Fl/Fl mice significantly increased the expression of FUT8, collagen III, p16^ink4a^, and p21^WAF1^, while decreased E-cadherin protein expression compared to saline-injected Fl/Fl mice (Figure 6B–6G). Knockout of FUT8 in CKO mice, on the other hand, reduced BLM-stimulated increased in the level of FUT8, as well as collagen III, p16^ink4a^, and p21^WAF1^ proteins expressions, and restored BLM-stimulated decreased in the expression of E-cadherin (Figure 6B–6G). Immunofluorescence and SA-β-gal staining further revealed that FUT8 knockout in CKO mice attenuated BLM-stimulated increased the fluorescence intensity of collagen I, p16^ink4a^, and p21^WAF1^ (Figure 6H–6K), as well as the number of positive cells for SA-β-gal activity (Figure 6L). Additionally, RT-PCR analysis suggested that FUT8 deletion increased the level of E-cadherin mRNA, and decreased FUT8 mRNA (Figure 6M) while decreasing the accumulation of hydroxyproline (Figure 6N). In conclusion, these results provided strong evidence that CF plays an extremely important role in pulmonary fibrosis. Blocking CF modification inhibits BLM-induced cell senescence and ameliorates lung fibrosis *in vivo*.

**Figure 7:**
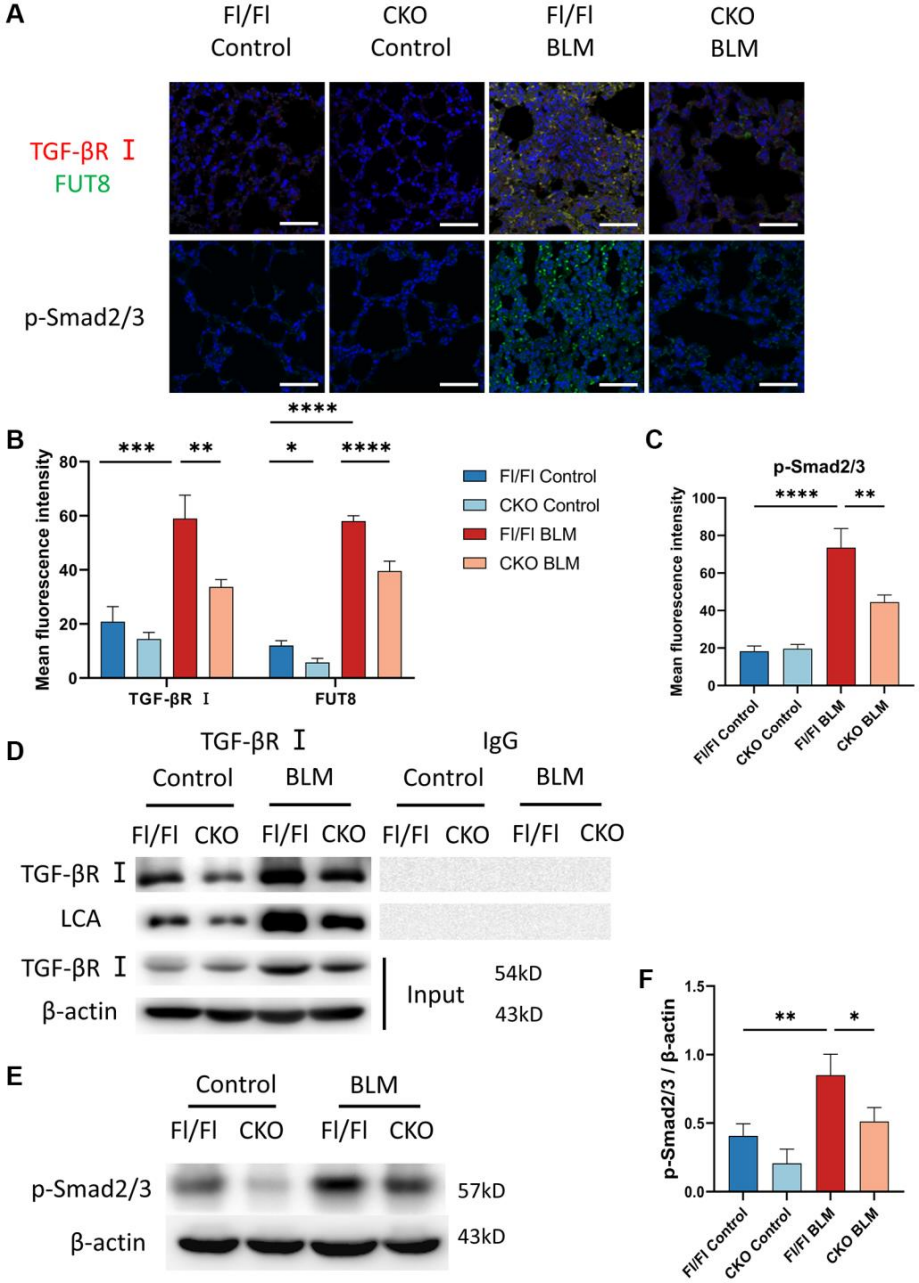
**Knockout CF modification of TGF-βR I inhibits activation of TGF-β signaling pathway *in vivo*.** (**A**–**C**) Representative immunofluorescence shows colocalization of TGF-βR I (Red) and FUT8 (Green) in different mice. The p-Smad2/3 expression in different treated mice was measured via immunofluorescence technique. Scale bar, 50 μm. Data were presented as the mean ± SD. ^*^P < 0.05. ^**^P < 0.01. ^***^P < 0.001. ^****^P < 0.0001. (**D**) Immunoprecipitation of TGF-βR I with LCA was tested by immunoblotting analysis in different mice. (**E**, **F**) Western blotting using anti-p-Smad2/3 and anti-β-actin antibodies in different treated mice. Densitometric analyses of the bands were adjusted to be equal to β-actin. Data were presented as the mean ± SD. ^*^P < 0.05. ^**^P < 0.01.

To assess whether CF regulates the TGF-β signaling pathway during AECs senescence *in vivo*, we measured CF modification of TGF-βR I and evaluated the expression of the downstream signaling pathway Smad2/3 phosphorylation in lung tissue. Indeed, immunofluorescence staining indicated that the fluorescence intensity of TGF-βR I and FUT8 increased in BLM-induced Fl/Fl mice while blocking FUT8 reduced the expression of TGF-βR I and FUT8 in BLM-induced CKO mice (Figure 7A, 7B). Furthermore, immunoprecipitation showed that LCA expression was lower in BLM-induced CKO mice than in BLM-induced Fl/Fl mice (Figure 7D). In addition, western blotting (Figure 7E, 7F) and immunofluorescence staining (Figure 7A, 7C) suggested that the phosphorylation level of Smad2/3 was increased in BLM-induced Fl/Fl mice compared with saline-injected Fl/Fl mice, but significantly decreased the p-Smad2/3 expression was observed in BLM-induced CKO mice compared with BLM-induced Fl/Fl mice. In conclusion, these data are consistent with the results of MLE_12_ cells *in vitro*.

## DISCUSSION

**Figure 8:**
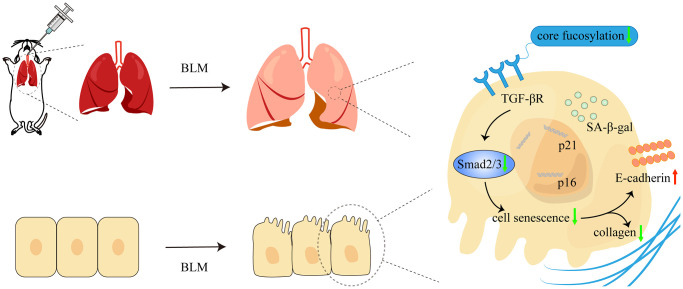
**The schematic diagram illustrated how core fucosylation regulated alveolar epithelial cells senescence in pulmonary fibrosis through TGF-β/Smad2/3 pathway *in vivo* and *in vitro*.** Blocking core fucosylation modification of TGF-βR could reduce the activation of the downstream pathways of TGF-β, obstructing the process of AECs senescence and partially ameliorating lung fibrosis.

Numerous lung diseases ultimately lead to pulmonary fibrosis, but its pathogenesis is still unknown and the prognosis is poor. The pathogenic mechanism of IPF is mediated by a complicated interaction between a number of signaling channels and several types of cells [30]. Evidence research indicated that AECs injury can occur in environmental, epigenetic, and other predisposing factors, resulting in senescence or aberrant epithelial cell activation [6, 30]. In our study, we confirmed that cellular senescence occurs in the BLM-induced mouse pulmonary fibrosis model. Furthermore, core fucosylation regulates alveolar epithelial cells senescence via activating of TGF-β signaling pathway in pulmonary fibrosis both *in vivo* and *in vitro* (Figure 8). These results indicated that CF modifications especially contribute to AECs senescence in lung fibrosis.

Relative studies have reported that FUT8 was strongly associated with lung disease [21, 31–33]. For example, in non-small cell lung cancer, FUT8 expression is up-regulated and is associated with tumor metastasis and a reduced prognosis for patients with non-small cell lung cancer [31]. Similarly, the development of an invasive and malignant tumor microenvironment was facilitated by FUT8-mediated CF in fibroblasts connected with cancer, and the ensuing non-small cell lung cancer cells showed accelerated proliferation and increased invasiveness [21]. Furthermore, upregulation of sialylation and fucosylation may reduce EGFR-mediated lung cancer cell invasion [32]. In lung tissues with COPD, collagen loss and reduction in CF were detected [33]. In this study, we implied that CF modifications accompany AECs senescence in pulmonary fibrosis. Both gain- and loss- of function research on CF were performed to elucidate its role in promoting AECs senescence and triggering pulmonary fibrosis *in vitro*. We found that silencing CF modifications with FUT8 siRNA ameliorated BLM-stimulated increase in FUT8, p16^ink4a^, and p21^WAF1^ protein expression and SA-β-gal activity, as well as collagen III and collagen I expression, and restores BLM-mediated suppression of E-cadherin. On the other hand, overexpression of FUT8 enhanced p16^ink4a^ and p21^WAF1^ proteins, which is linked to an increase in SA-β-gal activity. Moreover, our data also suggested, for the first time, using alveolar epithelial cell-specific FUT8 conditional knockout (CKO) mouse models, however, inhibition of cellular senescence by eliminating the FUT8 gene could attenuate pulmonary fibrosis *in vivo*.

TGF-β has long been identified as closely related to fibrotic disease [34]. TGF-β/Smad is a crucial route to control damage-induced and developmental senescence [35]. In a senescence paradigm, TGF-β1/MEK signaling promotes senescence-related pulmonary fibrosis [36]. What’s more, in lung fibrosis, pericyte transition was successfully prevented by inhibiting the CF of TGF-βR I [24]. Consistently with the reports, we verified that TGF-βR I can be modified by CF based on immunoprecipitation analysis. Silencing FUT8 reduced the BLM-mediated increase in TGF-βR I and LCA expression accompanied by a decrease in the p-Smad2/3 expression *in vivo* and *in vitro*. These results implied that blocking CF modification of TGF-βR I could reduce the activation of downstream pathways of TGF-β in AECs senescence. Similar to our findings, a recent study employed activating PI3K/AKT/IGF1 pathway depending on CF in AECs senescence [37]. Nevertheless, their work was devoted to examining the CF modifications primarily *in vitro* approaches and they didn’t use the genetic mouse models to demonstrate the role of CF in IPF more visually *in vivo*. In addition, because of TGF-β’s pleiotropic involvement in addressing a diverse set of downstream regulators, the mechanism underlying pulmonary fibrosis activation of TGF-β action has been associated with WNT crosstalk [27, 38], however, it has to be seen whether CF can activate the relevant signaling cascade by influencing other proteins such as EGFR to TGF-βR I.

Senescent cells release a variety of bioactive molecules, including proteases, chemokines, and profibrotic and proinflammatory cytokines. Which are referred to as the senescence-associated secretory phenotype (SASP) [17, 39]. Although we did not do a systematic study to dissect changes in SASP during AECs senescence, our preliminary studies showed that cellular senescence existed along with collagen deposition in BLM-induced lung fibrosis. Moreover, Blocking CF modification inhibited BLM-induced cell senescence and reduced the accumulation of collagen fibers. To date, academic researchers have identified novel “senotherapeutic” strategies that could be broadly classified into two categories: senolytic, which means to eliminate senescent cells; senomorphics, which contain SASP inhibitors and prevent the negative cell-extrinsic effects of senescent cells [40]. Evidence study showed that employing a three-dimensional lung tissue culture ex vivo models of lung fibrosis, senolytic drugs lessen the burden of senescent AECs and attenuated fibrotic markers [41]. Targeting CF may be a novel idea as a candidate for a senolytic strategy that could help IPF treatment.

A limitation of the study is that since we knockout FUT8 from the beginning of the BLM-induced CKO model, it may not be asserted that silencing FUT8 only influences the fibrotic phase *in vivo* because the BLM-induced lung fibrosis mice model initially exhibits inflammation reaction prior to the fibrotic period. Furthermore, in this study, we found that CF can promote cellular senescence by stimulating the TGF-β signaling pathway, which results in lung fibrosis. However, we do not yet know how cell senescence results in lung fibrosis; the potential mechanism remains to be revealed.

In summary, CF regulates AECs senescence by activating the TGF-β pathway in pulmonary fibrosis. Furthermore, blocking CF modification contributes importantly to inhibiting AECs senescence and attenuating pulmonary fibrosis. CF may be a critical interventional option for pulmonary fibrosis in therapy.

## MATERIALS AND METHODS

### Animal care and mouse lung fibrosis mouse model

6–8 weeks old male C57/BL6 mice (20 g–30 g, *n* = 12) were acquired from the Dalian Medical University Laboratory Animal Center. 6–8 weeks old male alveolar epithelial cell-specific FUT8 conditional knockout (FUT8^flox/flox^; Sftpc^CRE^, CKO) mice (20 g–30 g, *n* = 12) and FUT8^flox/flox^ (Fl/Fl) mice (20 g–30 g, *n* = 12) were constructed by Beijing Biocytogen Co., Ltd. (Beijing, China). They could eat and drink freely and were kept at a constant temperature throughout a 12-hour light and dark cycle. While conducting the experiments, every effort was made to cause the animals as little pain as possible. Male C57/BL6 mice, CKO mice, and Fl/Fl mice were both divided at random among 2 groups: Control group (*n* = 6), and BLM-induced fibrosis (BLM) group (*n* = 6); CKO Control group (*n* = 6), and CKO BLM group (*n* = 6); Fl/Fl Control group (*n* = 6), and Fl/Fl BLM group (*n* = 6). An intratracheal injection of bleomycin (5 mg/kg, MB1039, Dalian Meilun, Dalian, China) or an equal amount of saline was given to mice. All mice were killed after 3 weeks of intratracheal injection. The right lung’s upper lobe was fixed in 4% paraformaldehyde. (P1110, Solarbio, Beijing, China), while the upper lobe of the left lung was embedded in an optimal cutting temperature compound (4583, Sakura Finetek, Torrance, CA, USA). The right lung and the remaining left lobe are under cryopreservation at −80°C.

### Cell culture

MLE_12_ cells were bought from American Type Culture Collection (ATCC, Manassas, VA, USA). They were developed in DMEM/F12 (C11330500BT, Gibco, Los Angeles, CA, USA), containing 8% Fetal Bovine Serum (10099, Gibco, Los Angeles, CA, USA) and 1% penicillin-streptomycin solution (15070063, Gibco, Los Angeles, CA, USA) and then incubated at 37°C CO_2_.

### Adenovirus administration and siRNA design

The complete CDS of the FUT8 gene (NM_001002289.1) or blank gene was inserted into ADV4-CMV/IRES-GFP adenovirus vector system (GenePharma Co., Ltd, Shanghai, China). Negative target control (NT-OE) and adenovirus particles overexpressing FUT8 (FUT8-OE) were used to infect MLE_12_ cells in 6-well plates.

FUT8 siRNAs (GenePharma Co., Ltd, Shanghai, China) were chemically synthesized to target the FUT8 gene. SiRNA was powdered and reconstituted to a 20 nM final concentration in DEPC-treated H_2_O. The siRNAs and Lipofectamine 3000 (L3000015, Invitrogen, Gaithersburg, MD, USA) were complexed and added to the MLE_12_ cells incubated for at least 6 h. The siRNA sequence is as follows: 5′-GCUACUGAUGAUCCUACUU dTdT-3′; 5′-AAGUAGGAUCAUCAGUAGC dTdT-3′.

### Histological examination

After being treated in formalin, paraffin-embedded lung tissues were cut into 5 μm slices. The paraffin sections were dewaxed with xylene I/II for 15 min each, and ethanol absolute I/II for 2 min each. After dewaxing, using hematoxylin (G2160, Solarbio, Beijing, China) and eosin (G1102, Solarbio, Beijing, China) to perform Hematoxylin-Eosin (HE) staining, then using ethanol for gradient dehydration and transparenting with xylene. After being sealed with neutral resin, the sections were photographed using a light microscope (DM2500, Leica Microsystems, Wetzlar, Germany). Dewaxed sections were colored using the Masson staining after being soaked for 25 min in potassium dichromate solution. After that, they performed a procedure just like before, subsequently toluidine blue staining for 20 min.

### Senescence associated beta-galactosidase (SA-β-gal) staining

The SA-β-gal staining kit (G1580, Solarbio, Beijing, China) generates dark blue compounds catalyzed by aging-specific β-galactosidase using X-Gal as a substrate. Mixing Reagent X-Gal Solution, β-Gal Stain Solution A, β-Gal Stain Solution B, and β-Gal Stain Solution C as dyeing operating in a 5:1:1:93 ratio. Frozen sections were fixed for 15 minutes by adding enough β-Gal fixative to completely envelop the tissue. The tissues were washed with 1× PBS (10099, Thermo Fisher Scientific, Waltham, MA, USA) 3 times for 5 min each and then added an appropriate amount of dyeing operating solution in the wet box at 37°C overnight. Cells were washed once with PBS and 1 ml β-Gal fixative was added to each pore for 15 minutes in 6-well plates. Washing cells was daone 3 times for 3 min each with 1× PBS and then was added 1 ml dyeing operating solution. When incubated at 37°C overnight, the 6-well plate was required to be closed with parafilm M laboratory film. Finally, they were viewed under an optical microscope (DM2500, Leica Microsystems, Wetzlar, Germany). To calculate the percentage of senescent cells, total and SA-galactosidase-positive cells were counted in three randomly selected microscopic areas for each condition (100× magnification).

### Western blotting

Total proteins were isolated from MLE_12_ cells or lung tissues using Radio immunoprecipitation assay lysis buffer (P0013B, Beyotime Biotechnology, Shanghai, China) with protease inhibitors (P1005, Beyotime Biotechnology, Shanghai, China). The BCA Protein Assay Kit (P0012, Beyotime Biotechnology, Shanghai, China) was used to calculate the concentration of proteins. Protein samples are placed on a polyvinylidene fluoride membrane (1620176, Bio-Rad, Hercules, CA, USA) after being separated by 10% sodium dodecyl sulfate-polyacrylamide gel electrophoresis. After blocking, using primary antibodies such as anti-E-Cadherin (ab76055, Abcam, Cambridge, UK), anti- FUT8 (sc-271244, Santa Cruz Biotechnology, Dallas, TX, USA), anti-LCA (B-1045, Vector Laboratories, Burlingame, CA, USA), anti-collagen III (22734-1-AP, Proteintech, Rosemont, IT, USA), anti-β-actin (TA-09, ZSGB-Bio, Beijing, China), anti-p-smad2/3 (ab254407, Abcam, Cambridge, UK), anti-TGF-βR I (ab235178, Abcam, Cambridge, UK), anti-p16 (ab270058, Abcam, Cambridge, UK) and anti-p21 (ab188224, Abcam, Cambridge, UK), membranes were treated overnight at 4°C. Then incubated with appropriate secondary antibody HRP-labeled Goat Anti-Rabbit IgG (ZB-2301, ZSGB-Bio, Beijing, China) or HRP-labeled Goat Anti-Mouse IgG (A0216, Beyotime Biotechnology, Shanghai, China). The membrane was then visualized by ECL kit (SW2040, Solarbio, Beijing, China) and shot using AnalytikJena VisionWorks systems (UVP GelSolo, Analytik Jena AG, Jena, Germany). Image J software (1.8.0, National Institute of Health, Bethesda, MD, USA) was used to calculate the relative protein level. Protein expression levels were normalized by β-actin expression.

### Immunofluorescence (IF)

Lung tissues were sliced into paraffin sections after being treated with 4% paraformaldehyde (P1110, Solarbio, Beijing, China). Xylene and alcohol were used to deparaffinize the sections. In citrate buffer (pH 6.0, 10 mM) (C1010, Solarbio, Beijing, China), high temperature antigen recovery was carried out in the microwave for 15 min. Sections were blocked with FastBlock blocking buffer (YT876, Baiaolaibo Technology, Beijing, China) for 20 minutes. The sections were incubated with primary antibodies overnight at 4°C, then incubated with Alexa Fluor 488 (A0423, Beyotime Biotechnology, Shanghai, China) or Alexa Fluor 555 (A0460, Beyotime Biotechnology, Shanghai, China) for 1 h. DAPI (C1005, Beyotime Biotechnology, Shanghai, China) was used to stain the nuclei, then slices were mounted with glycerol for evaluation. Finally, slices were photographed at 200× magnification using a confocal microscope (SP8, Leica Microsystems, Wetzlar, Germany).

Cells cultured in 12-well plates (712001, Nest, Jiangsu, China) were first fixed for 25 min with 4% paraformaldehyde, followed by three washes of phosphate buffered saline, and permeabilized for 20 min with 1% Triton X-100 (P1080, Solarbio, Beijing, China). After blocking with FastBlock blocking buffer (YT876, Baiaolaibo Technology, Beijing, China) for 20 min, the procedure that followed was equivalent to that of tissue sections.

Using primary antibodies such as anti-LCA (B-1045, Vector Laboratories, Burlingame, CA, USA), anti-FUT8 (sc-271244, Santa Cruz Biotechnology, Dallas, TX, USA), SPC (sc-518029, Santa Cruz Biotechnology, Dallas, TX, USA) anti-collagen I (ab270993, Abcam, Cambridge, UK), anti-E-Cadherin (ab76055, Abcam, Cambridge, UK), anti-p-smad2/3 (ab254407, Abcam, Cambridge, UK), anti-TGF-βR I (ab235178, Abcam, Cambridge, UK), anti-p16 (ab270058, Abcam, Cambridge, UK), anti-p21 (ab188224, Abcam, Cambridge, UK), and anti-collagen III (22734-1-AP, Proteintech, Rosemont, IL, USA).

### FUT8 enzyme activity assay

The previous technique was used to evaluate the activity of FUT8 enzymes [42]. Donor (500 μM GDP-L-Fucose), assay buffer (200 mM MES), and the substrate (50 μM gngn-asn-4-(2-pyridylamine) butylamine (paba)) received five micrograms of cell lysates as an additional supply of enzymes. After incubation at 37°C about for 6 h, the mixture was heated at 100°C to halt the reaction for 5 min. The reactive solutions were centrifuged at 12,000 g for 10 min and then submitted to high performance liquid chromatography (Nexera LC-40, Shimadzu, Kyoto, Japan).

### Real-time polymerase chain reaction (RT-PCR)

To extract total RNA from lung tissues or cells, trizol reagent (R1100, Solarbio, Beijing, China) was employed. RNA was transformed into cDNA by means of reverse transcriptase kits (AG11728, Accurate Biology, Changsha, China). Using a 20 μl SYBR-Green PCR system, RT-PCR was carried out on Roche LightCycler^®^96 (AG11733, Accurate Biology, Changsha, China). Applying the 2^−ΔΔCT^ approach for relative quantification, data analysis was carried out. The levels of the gene were estimated relative to the β-actin gene. Primer sequences are followed:

**Table:** 

*E-cadherin*	F: 5′-GACAGGCTGGCTGAAAGTGA-3′
	R: 5′-CTTCATCACGGAGGTTCCTGG-3′
*FUT8*	F: 5′-AATACTTGATTCGTCCACAAC-3′,
	R: 5’-CTTCTGTTCCCACTTTGTCTG-3′;
*β-actin*	F: 5′-CATCCGTAAAGACCTCTATGCCAAC-3′,
	R: 5′-ATGGAGCCACCGATCCACA-3′.

### Immunoprecipitation

Protein lysates (500 μg) were incubated with anti-TGF-βR I (sc-518086, Santa Cruz Biotechnology, Dallas, TX, USA) and normal rabbit IgG (A7016, Beyotime Biotechnology, Shanghai, China) for 4-8 h, followed by adding 20 μl protein A/G plus-agarose (sc-2003, Beijing, China, Dallas, TX, USA) overnight at 4°C on an oscillation machine. The immunoprecipitates were washed with lysis buffer and centrifuged at 1000 g at 4°C three times for 3 min each. Equal amounts of proteins were tested by western blotting analysis.

### Hydroxyproline measurement

After being divided into smaller pieces, lung tissues were put into a 1.5 ml EP tube and acid hydrolyzed for 4 hours at 110°C. The solutions were centrifuged at 16,000 rpm for 20 minutes at 25°C after being neutralized with sodium hydroxide. According to the manufacturer's instructions, the hydroxyproline detection kit (BC0250, Solarbio, Beijing, China) was used to determine the amount of hydroxyproline contained in the collected supernatants.

### Statistical analysis

Statistical analysis was performed using GraphPad Prism v.9.0 software (GraphPad Software Inc., San Diego, CA, USA). The student’s *t*-test was used to compare two groups. A one-way analysis of variance (ANOVA) was used to compare the means of various groups. Data are presented as mean ± standard deviation (SD). Significance was considered as *P* < 0.05.
